# Artemisinin alleviates arsenic-induced myocardial injury in rats by modulating oxidative stress and inflammatory responses: Artemisinin alleviates arsenic-induced myocardial injury

**DOI:** 10.3724/abbs.2024225

**Published:** 2024-12-23

**Authors:** Wenjuan Qin, Yifei Zhou, Chuncui Chen, Xueting Guo, Ruimeng Tian, Ruoxi Chen, Wenrong Shi, Lei Huang, Caiyun Zhang, Shanshan Dong, Guilin Lu

**Affiliations:** Department of Ultrasonography the First Affiliated Hospital Shihezi University Shihezi 832008 China

Arsenic is widely present in nature, and its compounds are extensively used in industrial, agricultural, and medical fields [1]. Arsenic trioxide (As _2_O _3_) is specifically used as a therapeutic agent for acute promyelocytic leukemia because it induces cancer cell differentiation and apoptosis, significantly reduces the cancer cell count and has unique medical value [2]. However, owing to its high toxicity and carcinogenicity, long-term use can induce cardiovascular diseases such as arrhythmia and myocardial contractile dysfunction [3]. However, research on the treatment of arsenic-induced cardiotoxicity remains relatively scarce. Notably, artemisinin has anti-inflammatory and antioxidative effects on various heart diseases, effectively inhibiting reactive oxygen species (ROS) production, preventing myocardial damage and apoptosis caused by arsenic poisoning, and improving cardiac contractile and diastolic functions, thus enhancing cardiac function [4]. This study aims to discuss the impact of artemisinin on the myocardium of arsenic-poisoned rats.

Forty 12-week-old male SD rats [provided by SPF (Beijing) Biotechnology Co. Ltd, Beijing, China; SCXK-2024-0010] were randomly divided into five groups (8 rats per group): the control group (Con group), arsenic poisoning group (As group), drug control group (NC group, 30 mg/kg), low-dose artemisinin group (Art-L group, 30 mg/kg), and high-dose artemisinin group (Art-H group, 60 mg/kg). As _2_O _3_ was intraperitoneally injected at 5 mg/kg/day for 10 days in the arsenic poisoning group, low-dose group, and high-dose group, whereas the control group and drug control group received equal volumes of physiological saline. Artemisinin was subsequently injected at the corresponding doses for three weeks. After the rat model was established, myocardial contrast images were obtained via the EPIQ7C ultrasound system (Philips, Eindhoven, Netherlands) and loaded into the QLab 12.0 workstation to obtain the time to peak (TTP), peak intensity (PI), wash-in slope (WIS), and area under the curve (AUC). Myocardial perfusion blood flow was represented by WIS×PI, with values measured three times and averaged. Blood samples were collected from the inner canthus vein, and myocardial injury marker and inflammatory factor levels were determined via kits from Nanjing Jiancheng Bioengineering Institute (Nanjing, China). Rats were then sacrificed to obtain myocardial tissue samples, and myocardial peroxidase levels were measured according to the instructions of superoxide dismutase (SOD) and reduced glutathione (GSH) kits. Myocardial tissue samples were fixed, embedded, sectioned, stained, and observed under an electron microscope to collect relevant images. CD31 expression in myocardial cells was measured via immunofluorescence technology. All animal experiments were conducted according to the “Guidelines and Operational Procedures for Experimental Animals” approved by the Experimental Animal Ethics Committee of the First Affiliated Hospital of Shihezi University School of Medicine.

Myocardial contrast echocardiography (MCE) is a non-invasive method for assessing myocardial blood perfusion and is an effective tool for monitoring myocardial microcirculation [5]. Compared with the control and drug control groups, the As group presented a significantly lower AUC and WIS × PI, indicating decreased myocardial blood perfusion ability. With artemisinin intervention, the AUC and WIS × PI values increased to a certain extent, more notably at higher concentrations. This is due to myocardial cell damage and cardiac dysfunction induced by arsenic poisoning, coupled with increased myocardial oxygen consumption from tachycardia and increased cardiac output caused by inflammation. Artemisinin antagonizes this reaction, effectively alleviating myocardial ischemia ( Table 1).

**Table:** **Table 1** MCE parameters of each group of rats

	Con	NC	As	Art-L	Art-H	F	*P*
WIS (dB/s)	28.32 ± 4.28	27.92 ± 3.34	5.55 ± 1.10*	10.55 ± 3.11* ^,+^	15.67 ± 3.01* ^,+,#^	84.93	< 0.05
PI (dB)	128.67 ± 3.73	127.84 ± 5.83	19.14 ± 4.67*	46.47 ± 5.83* ^,+^	79.81 ± 3.79* ^,+,#^	802.15	< 0.05
WIS×PI (dB ^2^/s)	3477.89 ± 215.52	3506.05 ± 203.72	136.95 ± 81.47*	517.97 ± 209.46* ^,+^	1259.54 ± 233.91* ^,+,#^	539.68	< 0.05
TTP (s)	13.05 ± 3.10	13.44 ± 3.03	14.37 ± 3.36	13.08 ± 2.18	13.65 ± 3.85	0.24	0.92
AUC	2746.62 ± 179.03	2724.73 ± 196.43	427.83 ± 148.36*	844.48 ± 91.74* ^,+^	1301.52 ± 189.95* ^,+,#^	336.38	< 0.05

* *P*  < 0.05 compared with the Con and NC groups; ^+^ *P*  < 0.05 compared with the As group; ^#^ *P*  < 0.05 compared with the Art-L group. PI: peak intensity; WIS: wash-in slope; AUC: area under the curve; TTP: time to peak.

CD31 is a specific vascular endothelial marker that is positively correlated with myocardial angiogenesis and is often used to assess microvascular density [6]. In this study, the control and drug control groups presented lower CD31 (green) expression in myocardial cells. The As group exhibited extensive myocardial cell damage, oxidative reactions, and inflammation, promoting microvascular formation and resulting in significant fluorescence reactions. After intervention with different concentrations of artemisinin, the fluorescence reactions weakened to varying degrees with increasing concentrations, indicating the protective effect of artemisinin on myocardial cell damage induced by arsenic ( Figure 1A,B).

**Figure 1:**
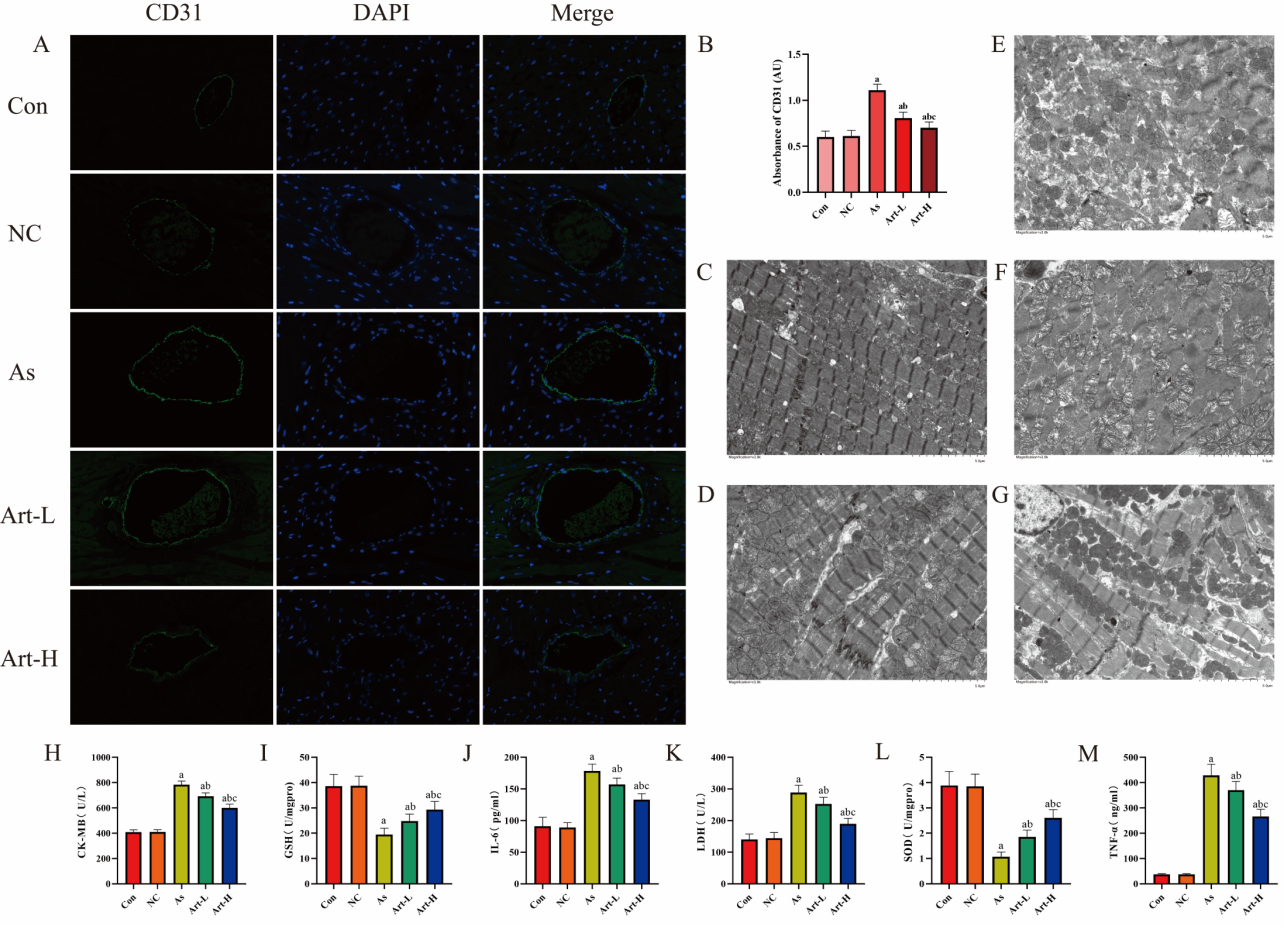
Figure 1 The effects of artemisinin on contrast-enhanced ultrasound, myocardial tissue structure, and biochemical markers in arsenic-poisoned rats (A) The effect of artemisinin on the immunofluorescence staining of myocardial tissue in arsenic-poisoned rats CD31 (green) and DAPI (blue). Magnification, ×400. (B) CD31 immunofluorescence absorbance values in each group of rats. (C–G) Electron microscopy results of myocardial tissue in rats treated with artemisinin. (C) Con group. (D) NC group. (E) As group. (F) Art-L group. (G) Art-H group. (H–M) Blood serum samples were collected from each group to measure the following parameters: (H) CK-MB, creatine kinase-MB; (I) LDH, lactate dehydrogenase; (J) GSH, glutathione; (K) SOD, superoxide dismutase; (L) IL-6, interleukin-6; and (M) TNF-α, tumor necrosis factor-α. a P < 0.05 compared to the Con and NC groups; b P < 0.05 compared to the As group; c P < 0.05 compared to the Art-L group.

Additionally, electron microscopy results were analyzed to verify the experimental conclusions [7]. The control and drug control groups presented clear myocardial myofibrils, neatly arranged myocardial cells with clear contours and full shapes, and no noticeable necrotic cells. The As group presented disordered myocardial myofibrils with many fractures, separated myofilament bundles, tortuous Z-line structures, no H bands, myocardial cells of varying sizes and shapes, some shrunken and dissolved nuclei, swollen and enlarged mitochondria, irregular shapes, increased autophagic bodies, and various degrees of endoplasmic reticulum shrinkage. In the low-dose and high-dose groups, there was some improvement in the myofibrils, myocardial cells, and organelles, which became more pronounced with increasing artemisinin dosage ( Figure 1C–G).

Previous studies indicated that arsenic exposure leads to cardiac lipid peroxidation, generating large amounts of inflammatory factors, mediating oxidative stress, reducing myocardial contractility, and affecting the extracellular matrix, leading to endoplasmic reticulum stress (ERS) imbalance and inducing apoptosis [8]. The endoplasmic reticulum is crucial for protein synthesis, folding, and processing [9]. Arsenic poisoning causes excessive ROS formation, disrupting the oxidative-antioxidative system balance. High ROS levels significantly induce protein misfolding in the endoplasmic reticulum, ultimately causing tissue damage. SOD and GSH effectively reflect myocardial peroxidation [10], and the trends in immunofluorescence, contrast echocardiography, and myocardial injury markers (CK-MB, LDH, TNF-α, and IL-6) and inflammatory factors were consistent with the SOD and GSH results. Thus, artemisinin can reduce myocardial damage caused by inflammation in a dose-dependent manner ( Figure 1H–M).

In conclusion, artemisinin can inhibit ROS production, maintain extracellular matrix homeostasis, counteract inflammation and oxidative stress, and reduce myocardial cell damage caused by arsenic poisoning. This study provides insights for treating arsenic poisoning patients, but further clinical applications need to be explored.

## Supporting information

24576Table_1
