# Supplementary material for: Artemisinin alleviates arsenic-induced myocardial injury in rats by modulating oxidative stress and inflammatory responses: Artemisinin alleviates arsenic-induced myocardial injury
Source: Acta Biochim Biophys Sin (Shanghai). 2024 Dec 23;57(6):1033–5. doi: 10.3724/abbs.2024225 (PMC12247132; doi:10.3724/abbs.2024225)
Supplement: 24576Table_1 [file 24576Table_1.docx]

**Table 1. MCE parameters of each group of rats**

|  | Con | NC | As | Art-L | Art-H | F | *P* |
| --- | --- | --- | --- | --- | --- | --- | --- |
| WIS (dB/s) | 28.32±4.28 | 27.92±3.34 | 5.55±1.10* | 10.55±3.11*^,+^ | 15.67±3.01*^,+,#^ | 84.93 | <0.05 |
| PI (dB) | 128.67±3.73 | 127.84±5.83 | 19.14±4.67* | 46.47±5.83*^,+^ | 79.81±3.79*^,+,#^ | 802.15 | <0.05 |
| WIS×PI (dB^2^/s) | 3477.89±215.52 | 3506.05±203.72 | 136.95±81.47* | 517.97±209.46*^,+^ | 1259.54±233.91*^,+,#^ | 539.68 | <0.05 |
| TTP (s) | 13.05±3.10 | 13.44±3.03 | 14.37±3.36 | 13.08±2.18 | 13.65±3.85 | 0.24 | 0.92 |
| AUC | 2746.62±179.03 | 2724.73±196.43 | 427.83±148.36* | 844.48±91.74*^,+^ | 1301.52±189.95*^,+,#^ | 336.38 | <0.05 |

**P*<0.05 compared with the Con and NC groups; ^+^*P*<0.05 compared with the As group; ^#^*P*<0.05 compared with the Art-L group. PI: peak intensity; WIS: wash-in slope; AUC: area under the curve; TTP: time to peak.
